# Combination of Lycopene and Curcumin Synergistically Alleviates Testosterone-Propionate-Induced Benign Prostatic Hyperplasia in Sprague Dawley Rats via Modulating Inflammation and Proliferation

**DOI:** 10.3390/molecules28134900

**Published:** 2023-06-21

**Authors:** Shanshan Wang, Wenjiang He, Wenzhi Li, Jin-Rong Zhou, Zhiyun Du

**Affiliations:** 1 School of Biomedical and Pharmaceutical Sciences, Guangdong University of Technology, Guangzhou 511400, China; wangs@mail2.gdut.edu.cn; 2 R&D Centre, Infinitus (China) Company Ltd., Guangzhou 510520, China; kenny.he@infinitus-int.com (W.H.); peter.wenzhi.li@infinitus-int.com (W.L.); 3 Nutrition/Metabolism Laboratory, Department of Surgery, Beth Israel Deaconess Medical Center, Harvard Medical School, Boston, MA 02215, USA; 4 Conney Allan Biotechnology Company Ltd., Guangzhou 510095, China; 5 Institute of Biomedical and Pharmaceutical Sciences, Guangdong University of Technology, Guangzhou 511400, China

**Keywords:** BPH, lycopene, curcumin, synergistic effect, inflammation

## Abstract

Background: Benign prostatic hyperplasia (BPH) is a progressive urological disease occurring in middle-aged and elderly men, which can be characterized by the non-malignant overgrowth of stromal and epithelial cells in the transition zone of the prostate. Previous studies have demonstrated that lycopene can inhibit proliferation, while curcumin can strongly inhibit inflammation. This study aims to determine the inhibitory effect of the combination of lycopene and curcumin on BPH. Method: To induce BPH models in vitro and in vivo, the BPH-1 cell line and Sprague Dawley (SD) rats were used, respectively. Rats were divided into six groups and treated daily with a vehicle, lycopene (12.5 mg/kg), curcumin (2.4 mg/kg), a combination of lycopene and curcumin (12.5 mg/kg + 2.4 mg/kg) or finasteride (5 mg/kg). Histologic sections were examined via hematoxylin and eosin (H&E) staining and immunohistochemistry. Hormone and inflammatory indicators were detected via ELISA. Network pharmacology analysis was used to fully predict the therapeutic mechanism of the combination of lycopene and curcumin on BPH. Results: Combination treatment significantly attenuated prostate hyperplasia, alleviated BPH pathological features and decreased the expression of Ki-67 in rats. The upregulation of the expression of testosterone, dihydrotestosterone (DHT), 5α-reductase, estradiol (E2) and prostate-specific antigen (PSA) in BPH rats was significantly blocked by the combination treatment. The expression levels of inflammatory factors including interleukin (IL)-1β, IL-6 and tumor necrosis factor (TNF)-α were strongly inhibited by the combination treatment. From the network pharmacology analysis, it was found that the main targets for inhibiting BPH are AKT1, TNF, EGFR, STAT3 and PTGS2, which are enriched in pathways in cancer. Conclusion: The lycopene and curcumin combination is a potential and more effective agent to prevent or treat BPH.

## 1. Introduction

Benign prostatic hyperplasia (BPH), the most common chronic disease among aging men [1], can potentially lead to lower urinary tract symptoms (LUTSs) [2,3,4]. Epidemiological studies show the incidence of BPH increases with age, which reaches 40–50% at the age of 50–60 and up to 80–90% after the age of 80 [5,6]. Histologically, BPH is characterized by the hyperproliferation of the glandular epithelium and supporting stromal cells of the prostate gland [7]. At the biochemical level, BPH is considered to be caused by the imbalance between androgen and estrogen and the over-expression of growth factors in stromal and epithelial cells [8].

Although the underlying mechanisms of BPH development are not fully understood, various pathogenic mechanisms have been proposed, including oxidative stress, inflammation, the imbalance of proliferation and apoptosis and hormonal imbalances [9]. Indeed, the androgenic pathway is thought to be predominant. Dihydrotestosterone (DHT), a powerful prostatic androgen, is converted from testosterone by 5α-reductase [10]. With a high affinity, DHT binds to the androgen receptor, which initiates the transcription of genes that encode differentiation and growth-promoting factors. This process subsequently stimulates prostatic proliferation [11]. Furthermore, inflammation is a prevalent observation in the prostate, evident on both histological and biochemical levels, thereby promoting the advancement of BPH [12]. The inflammation observed in BPH may result in tissue damage, and the cytokines generated by inflammatory cells may stimulate the production of growth factors and angiogenesis in the prostate, as a form of wound-healing response [13]. The major pathological feature of BPH is the hyperplasia of prostatic epithelial and stromal cells, caused by an imbalance between cell growth and apoptosis [14]. Apoptosis is programmed cell death which occurs on a regular basis to keep a homeostatic balance between cell proliferation and death rates [15].

The management of BPH typically entails the utilization of Alpha-1 adrenergic receptor blockers, 5α-reductase enzyme inhibitors (e.g., finasteride (FN)) and surgical intervention, or a combination of these therapeutic modalities [16]. Research has shown that different types of drugs have different side effects. Alpha-1 blockers can be associated with orthostatic hypotension, and 5α-reductase inhibitors are associated with sexual dysfunction [17,18].

Interestingly, natural plant extracts are emerging as an innovative modality to treat BPH and show significant improvement or even curative effects, and they have less side effects than common conventional medical treatments [19]. Lycopene (LY) is a natural pigment with anti-oxidant and anti-proliferative activities. Epidemiological studies have shown that high intake of LY is related to the reduced risk of prostatic diseases like BPH and prostatic cancer [20,21,22]. Zou et al. has found that 30 mg/kg Maca and 7.5 mg/kg LY can effectively inhibit the progression of BPH. However, the synergistic effect of these two drugs was not researched in this study [23]. Curcumin (CUR), derived from the rhizome of *Curcuma longa* L., is a medicinal herb with a long history of use in the Indian system of medicine for the treatment of various health conditions over many centuries [24]. CUR possesses a wide range of therapeutic effects such as anti-inflammatory, anti-oxidant, anti-cancer and anti-microbial [25,26,27]. There is a research suggesting that 50 mg/kg curcumin via daily oral administration showed an inhibitory effect on BPH in rats [28].

However, the utilization of a solitary plant extract for the treatment of BPH necessitates a higher dosage, which may impose a greater strain on the liver and kidney functions [29]. Furthermore, monotherapy typically targets a singular aspect, and prolonged usage may result in the development of drug resistance [30]. The implementation of combined therapy presents a compelling strategy for managing chronic ailments. As assessed using the International Prostate Symptom Score, combination therapy has demonstrated an enhanced likelihood of better recovery and response rates, while also mitigating drug resistance in patients with long-standing chronic conditions [30]. Therefore, this study aims to evaluate the potential synergistic effects of co-administering LY and CUR in mitigating the development of BPH in rats, while also gaining insight into the multi-target mechanism underlying this combined therapeutic approach.

## 2. Results

### 2.1. Effects of LY and CUR on the Viability of BPH-1 Cells In Vitro

**Figure 1:**
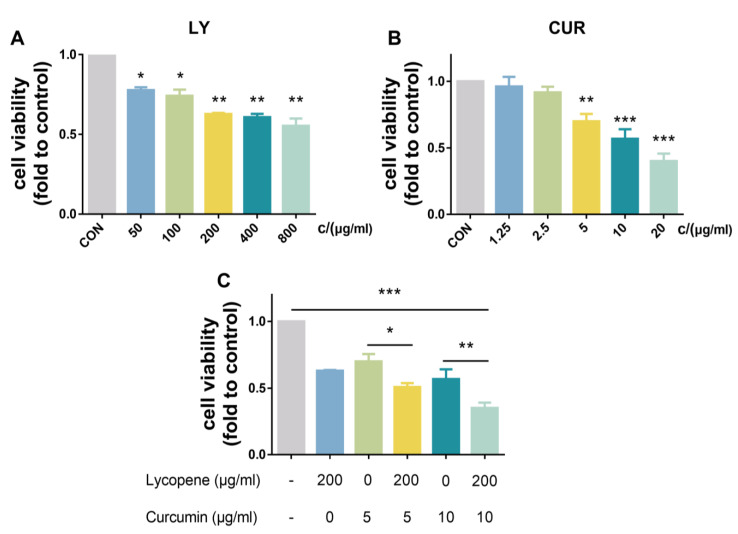
Effects of LY and CUR on the viability of BPH-1 cells. (**A**) The dose-dependent effect of LY on cell viability; (**B**) the dose-dependent effect of CUR on cell viability; (**C**) the effect of LY/CUR combinations on cell viability. All values are means ± SD (*n* = 6). Within each panel, the values with the superscription symbol are significantly different from that of the control; * *p* < 0.05, ** *p* < 0.01 and *** *p* < 0.001.

The effects of LY, CUR and a combination of LY and CUR on the viability of BPH-1 cells were detected via a CCK-8 assay. As shown in Figure 1, LY (Figure 1A) and CUR (Figure 1B) inhibited the cell proliferation in a dose-dependent manner, and when the inhibition rate of cell proliferation reached about 50%, the concentrations of LY and CUR were 800 μg/mL and 10 μg/mL, respectively.

Since CUR showed more sensitive dose-dependent activity than LY, the lower doses of LY (200 μg/mL) and CUR (5, and 10 μg/mL) were selected for evaluating the combination effect, and the nature of the combination was determined by calculating the CI. As shown in Figure 1C, the LY and CUR combination had more potent activities than the corresponding individual treatment. Compared with the control, LY (200 μg/mL), CUR (5 μg/mL), CUR (10 μg/mL), the LY (200 μg/mL)/CUR (5 μg/mL) combination and the LY (200 μg/mL)/CUR (10 μg/mL) combination inhibited the BPH-1 cell viability by 37.19% (*p* < 0.001), 30.19% (*p* < 0.001), 43.23% (*p* < 0.001), 49.13% (*p* < 0.001) and 64.81% (*p* < 0.001), respectively. The CI for the LY (200 μg/mL)/CUR (10 μg/mL) combination treatment was 0.9 *<* 1. The results indicated that the LY and CUR combination had a synergistic effect on inhibiting the proliferation of BPH-1 cells.

### 2.2. Effects of LY and CUR Combination Treatments on BPH Development in Rats

**Figure 2:**
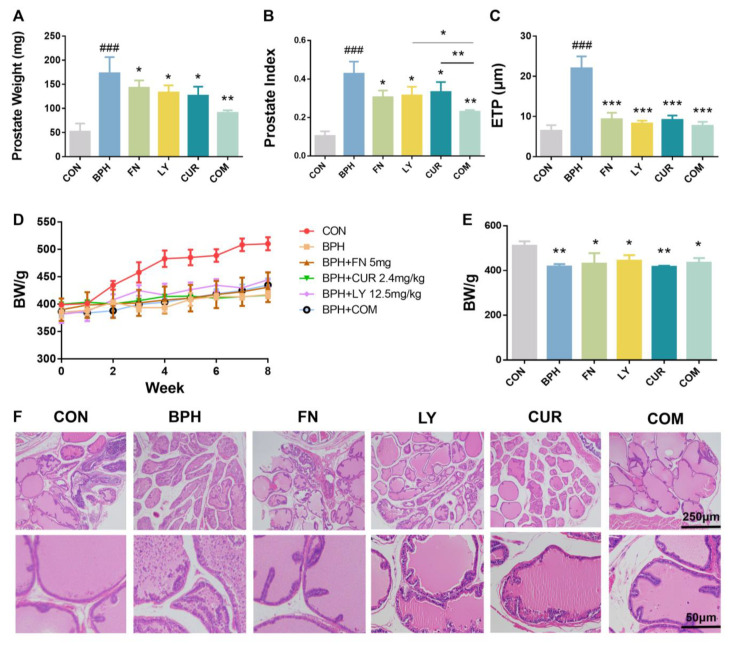
Effects of experimental treatments on BPH development in rats. (**A**) Prostatic weight; (**B**) prostate index; (**C**) quantified results of epithelium thickness of prostate (ETP); (**D**) time-dependent body weight change; (**E**) body weight of rats at week 8; (**F**) representative H&E staining images. All values are mean ± SD (*n* = 6). Within each panel (**A**–**C**), the values with the superscription symbols are significantly different from those of the corresponding control; * *p* < 0.05, ** *p* < 0.01 and *** *p* < 0.001, when compared with the BPH group; ### *p* < 0.001, compared with the CON group; one-way ANOVA followed by Tukey’s comparison test.

Rats were induced for BPH via the subcutaneous injection of TP and treated with FN or experimental drugs. After 8 weeks of induction with TP, both the prostatic weight (Figure 2A) and prostate index (prostate weight (mg)/body weight (g)) (Figure 2B) in the BPH group were significantly higher than those in the control group, indicating that the BPH model was successfully established. Of note, the TP-induced increases in prostate weight and prostate index were significantly attenuated by the LY and CUR treatments. The prostate weights of rats in the LY, CUR and COM groups were significantly reduced by 23.08% (*p* < 0.05), 26.78% (*p* < 0.05) and 47.35% (*p* < 0.01), compared with those in the BPH group (Figure 2A). In parallel, the prostate indices in the rats in the LY, CUR and COM groups were significantly reduced by 26.2% (*p* < 0.05), 22.18% (*p* < 0.05) and 46.26% (*p* < 0.01), compared with those in the BPH group (Figure 2B). The LY/CUR combination further increased the individual treatment effect, and the CI for the combination treatment was 0.229 *<* 1, suggesting that the LY/CUR combination may provide more a potent treatment regimen than individual treatments in inhibiting BPH development.

The epithelium thicknesses of the prostate (ETPs) were determined in the H&E-stained prostate tissues. The ETP in the BPH group was significantly increased by 241%, compared with that in the CON group (Figure 2C,F). Treatments with LY, CUR and the LY/CUR combination significantly reduced the ETP by 62.51% (*p* < 0.001), 58.45% (*p* < 0.001) and 65.16% (*p* < 0.001), respectively, compared with the BPH group.

To sum up, these data indicate that the combination of LY and CUR can inhibit the development of BPH and is more effective than LY and CUR alone.

### 2.3. Effects of LY and CUR Combination Treatments on Serum Levels of Hormones in Rats

**Figure 3:**
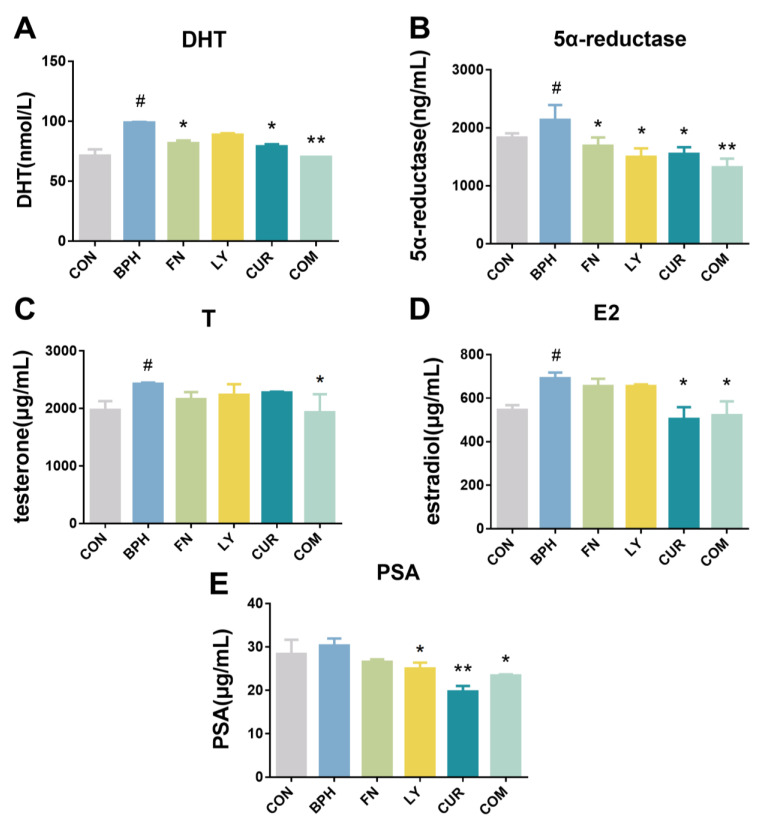
Effects of experimental treatments on serum levels of hormones. (**A**) Dihydrotestosterone (DHT), (**B**) 5α-reductase, (**C**) testosterone (T), (**D**) estradiol (E2) and (**E**) prostate-specific antigen (PSA) in rats. All values are mean ± SD (*n* = 6). # *p* < 0.05 vs. CON group, * *p* < 0.05, ** *p* < 0.01 vs. BPH group, one-way ANOVA followed by Tukey’s comparison test.

As shown in Figure 3A–D, the serum levels of DHT, 5α-reductase, T and E2 were indicated as being significantly higher in the BPH group by 1.37-, 1.17-, 1.23- and 1.27-fold compared with the CON group, suggesting TP administration had resulted in the upregulation of the serum level of DHT, 5α-reductase, T and E2 and the consequent benign prostate hyperplasia. Of note, treatments with LY, CUR and the LY/CUR combination significantly reduced the expression of DHT by 10.17%, 19.97% (*p* < 0.05) and 29.06% (*p* < 0.01), respectively, compared with the BPH group. The expression levels of 5α-reductase in the LY, CUR and COM group were significantly decreased by 29.87% (*p* < 0.05), 27.48% (*p* < 0.05) and 38.3% (*p* < 0.01), respectively, compared with the BPH group. Compared with the BPH group, the regulation of testosterone via LY and CUR was not significant, but the expression of T in the COM group was reduced by 20.35% (*p* < 0.05). The expression of E2 and PSA in the COM group was decreased by 24.58% (*p* < 0.05) and 22.77% (*p* < 0.05), respectively, compared with the BPH group.

### 2.4. Effects of LY and CUR Combination Treatments on Inflammatory Responses in Rats

**Figure 4:**
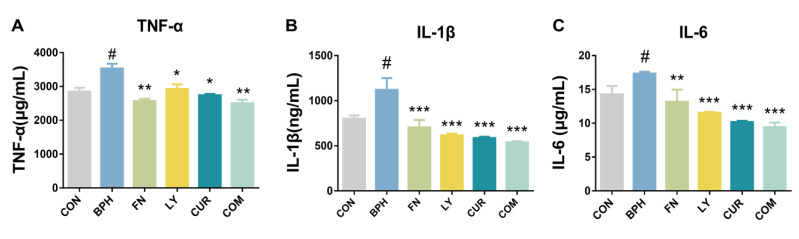
Assessment of treatment effects on serum levels of inflammatory cytokines TNF-α (**A**), IL-1β (**B**) and IL-6 (**C**). All values are mean ± SD (*n* = 6). # *p* < 0.05 vs. CON group, * *p* < 0.05, ** *p* < 0.01, *** *p* < 0.001 vs. BPH group, one-way ANOVA followed by Tukey’s comparison test.

As shown in Figure 4A–C, BPH induction stimulated the secretion of TNF-α, IL-1β and IL-6 into blood and significantly increased the serum levels by 1.24, 1.4 and 1.22 in comparison with the CON group. However, the TNF-α, IL-1β and IL-6 levels in the COM group were significantly lower than those in the BPH group by 29.13% (*p* < 0.01), 51.85% (*p* < 0.001) and 45.6% (*p* < 0.001), respectively.

### 2.5. Effects of LY and CUR Combination Treatments on Prostate Cell Proliferation

**Figure 5:**
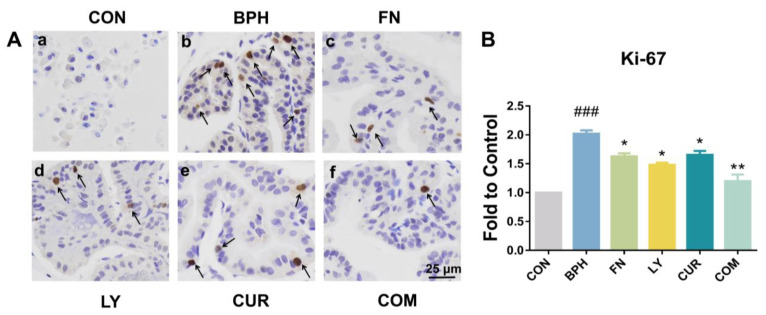
Immunohistochemistry (IHC) of prostatic tissues. (**A**) Animal prostates were collected and stained by cell proliferation marker Ki-67, in rats treated by (**a**) control group (CON), (**b**) BPH model group (BPH), (**c**) BPH+FN group (FN), (**d**) BPH+LY group (LY), (**e**) BPH+CUR group (CUR) or (**f**) BPH + LY + CUR group (COM), respectively. The arrows point to brown areas representing the expression of Ki-67. (**B**) Quantitative analysis of gray value was carried out by ImageJ. All values are mean ± SD (*n* = 3). ### *p* < 0.001 vs. CON group, * *p* < 0.05, ** *p* < 0.01 vs. BPH group, one-way ANOVA followed by Tukey’s comparison test.

To explore the inhibitory effect of drugs on proliferation, cell proliferation marker Ki-67 was detected. As shown in Figure 5, the expression level of Ki-67 in the BPH group was much higher than that in the CON group, as the gray value of the BPH group was twice that of the CON group. After treatment, the expression level of Ki-67 in the LY, CUR and COM group decreased by 17.27% (*p* < 0.05), 22.47% (*p* < 0.05) and 29.13% (*p* < 0.01), respectively, compared with the BPH group.

### 2.6. Network Pharmacology Analysis

**Figure 6:**
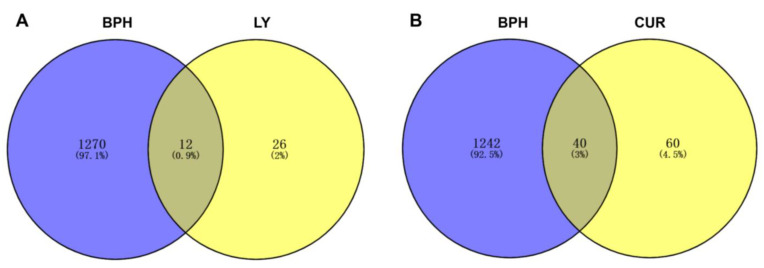
The predicted molecular candidate targets between the BPH- and LY-associated targets (**A**), or between the BPH- and CUR-associated targets (**B**).

The process of gathering and merging data pertaining to BPH-related targets from Targetnet, UniProt and Genecards yielded a total of 1282 targets that met the relevance threshold of greater than 6. A comparative analysis was conducted between these targets and the predicted targets of LY and CUR, resulting in the identification of 52 common targets that were deemed as potential candidates associated with the anti-BPH activity of LY (No. 1–12) and CUR (No. 13–52) (Table 1, Figure 6). Notably, there were no overlapping targets between LY and CUR, indicating that these compounds may impede the progression of BPH through the modulation of distinct targets.

**Figure 7:**
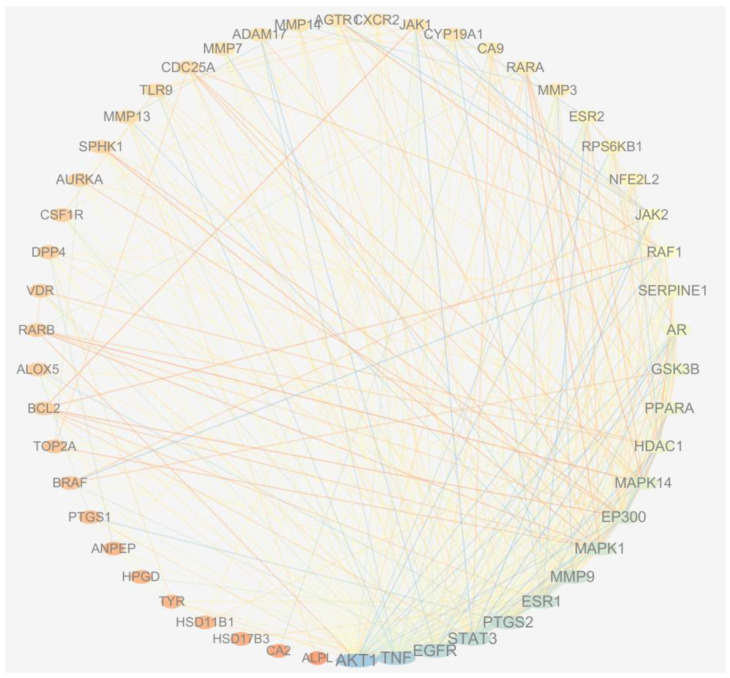
Protein–protein interaction network. The PPI network was constructed using Cytoscape and analyzed using NetworkAnalyzer. Different colors represent the degree. Node size is proportional to the degree of interaction.

In order to investigate the potential mechanisms underlying the synergistic effects of lycopene and curcumin against BPH, a total of 52 common targets were inputted into the STRING database to construct an original protein–protein interaction (PPI) network. The resulting network, consisting of 51 nodes and 339 edges, was generated from the tsv file (as shown in Figure 7). The analysis revealed five key targets, namely AKT1 (degree = 42), TNF (degree = 37), EGFR (degree = 33), STAT3 (degree = 32) and PTGS2 (degree = 30).

**Figure 8:**
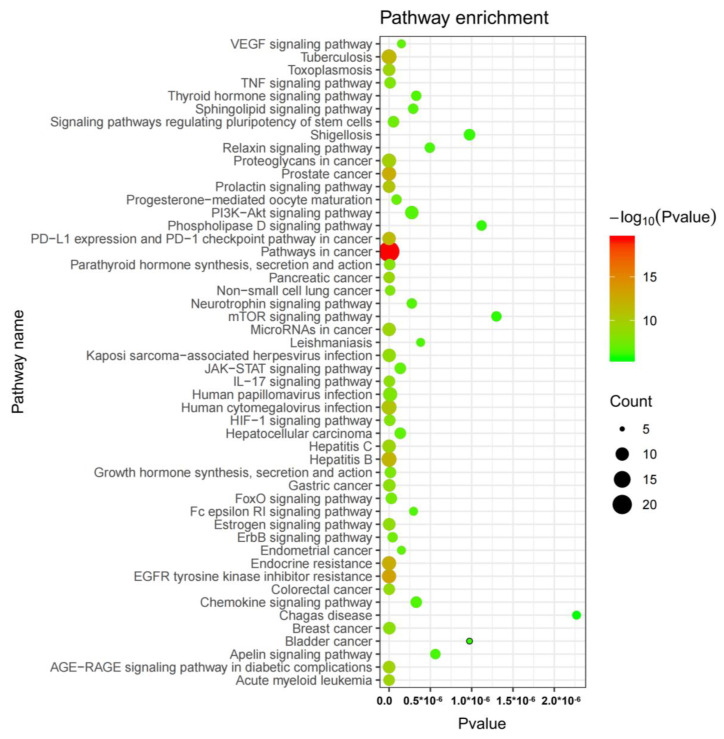
KEGG enrichment analysis of the anti-BPH targets of lycopene and curcumin.

A KEGG pathway enrichment analysis was conducted on the 52 common targets (Figure 8) with a significance level of *p* < 0.01. The results revealed the top three significantly enriched KEGG pathways, which were pathways in cancer (hsa05200), EGFR tyrosine kinase inhibitor resistance (hsa01521) and endocrine resistance (hsa01522).

## 3. Materials and Methods

### 3.1. Chemicals and Reagents

Testosterone propionate (TP) was purchased from Ningbo Second Hormone Factory (Ningbo, Zhejiang, China), and FN was purchased from Zhejiang CONBA Pharmaceutical Co., Ltd. (Hangzhou, Zhejiang, China). LY was purchased from Xinjiang Keyu Technology Co., Ltd. (Urumchi, Xinjiang, China). CUR was purchased from Yuanye Biotechnology Co., Ltd. (Shanghai, China). BPH-1 human benign prostatic hyperplasia cell line cells were purchased from Beina Biology (Beijing, China). ELISA kits for determining rat dihydrotestosterone (DHT), 5α-reductase, testosterone (T), estradiol (E2), prostate-specific antigen (PSA), interleukin (IL)-1β, IL-6 and tumor necrosis factor (TNF)-α were purchased from Jiangsu Meibiao Biotechnology Co., Ltd. (Yancheng, Jiangsu, China). Protein extraction solution was purchased from Solarbio Science & Technology Co., Ltd. (Beijing, China).

### 3.2. Cell Culture

Cell culture reagents were purchased from Gibco (Life Technologies, Gaithersburg, MD, USA) unless otherwise stated. The BPH-1 cells were cultured in DMEM medium supplemented with 10% (*v*/*v*) heat-inactivated FBS and 100 units/mL penicillin–streptomycin at 37 °C in a humidified atmosphere of 5% CO_2_.

### 3.3. Cell Viability Assay

Cell viability was assessed by using the Cell Counting Kit 8 (CCK-8) assay (Dojindo, Kumamoto, Japan). Briefly, BPH-1 cells (5 × 10^3^/well) were seeded in 96-well tissue culture plates and grown for 24 h. Cells in the treated groups were then treated with LY (50, 100, 200, 400 and 800 μg/mL), CUR (1.25, 2.5, 5, 10 and 20 μg/mL) and the LY/CUR combinations (200/5 and 200/10 μg/mL) for 24 h, being dissolved in the vehicle (DMEM medium). Cells in the control group were treated with the vehicle alone. The cell viability was measured via CCK-8 analysis following the instructions provided by the vendor. The experiments were triplicated and independently repeated at least twice.

### 3.4. Animal Study

Male Sprague Dawley rats (4 months old; 250–300 g), purchased from Guangdong Medical Laboratory Animal Center (Guangzhou, China), were housed with free access to food and water at the animal facility of South China University of Technology with a controlled environment of a 12 h light/dark cycle, temperature (22 ± 2 °C), and humidity (55 ± 9%). After one week of acclimatization, the rats were castrated and recovered for one week before BPH induction via the subcutaneous injection of TP (5 mg/kg BW, dissolved in 200 μL of olive oil) daily for 8 weeks (Figure 1). Rats in the control group were injected subcutaneously with olive oil alone. The animal study was reviewed and approved by the Animal Ethics Committee of South China University of Technology.

**Figure 9:**
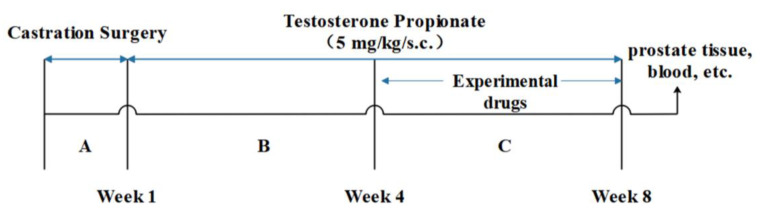
Experimental flow chart.

The castrated rats were randomly assigned into one of the following six experimental groups (*n* = 6/group) and received the corresponding treatment daily starting 4 weeks after the initiation of BPH induction (Table 2): (i) control group (CON): orally administered 1 mL of olive oil; (ii) BPH model group (BPH): administered orally with 1 mL of olive oil; (iii) BPH + FN group (FN): orally administered FN (5 mg/kg BW); (iv) BPH + LY group (LY): orally administered LY (12.5 mg/kg BW); (v) BPH + CUR group (CUR): orally administered CUR (2.4 mg/kg BW); (vi) BPH + LY + CUR group (COM): orally administered LY and CUR (12.5 + 2.4 mg/kg BW). After 28 consecutive days of treatments, the experiment was concluded. Subsequently, the rats were euthanized, and expeditiously, blood samples and prostates were extracted for subsequent analyses. A diagram of the experimental protocol is shown in Figure 9.

### 3.5. Histopathology and Immunohistochemistry (IHC)

The prostate tissues underwent fixation in 4% paraformaldehyde at 4 °C for 24 h, followed by dehydration and paraffin embedding and sectioning at a thickness of 5 µm. Hematoxylin and eosin (H&E) staining was utilized for histopathology, while immunohistochemistry (IHC) involved boiling the tissue slices in critic acid (pH 6.0) for 30 min for antigen retrieval, soaking them in 3% H_2_O_2_ for 10 min and blocking them with 2% normal goat serum (diluted 1:10 in PBS) for 30 min at 20 °C. The slices were then incubated with primary antibody overnight and secondary antibody for 30 min. Fields were selected using a systematic random sampling scheme [31]. Images were acquired using a light microscope (Nikon Eclipse E100, Tokyo, Japan), and the density was quantified by using ImageJ 1.50i.

### 3.6. Enzyme-Linked Immunosorbent Assay (ELISA)

The serum levels of DHT, T, E2, 5α-reductase, PSA, IL-1*β*, IL-6 and TNF-α were measured via ELISA following the protocols provided by the manufacturer.

### 3.7. Network Pharmacology

The gene targets of LY and CUR were identified through a comprehensive approach that involved the utilization of the Drugbank database, the Swisstarget Prediction database and the published literature. The target organism selected for this study was Homo sapiens. The BPH-associated gene targets were obtained from the Targetnet database, the UniProt database and the Genecards database. To establish a gene library of anti-BPH targets for LY and CUR, a comparative and analytical approach was employed to identify common targets between BPH-associated targets and the predicted targets of LY and CUR.

The collected BPH target library and the LY or CUR target library were analyzed using Venny (https://bioinfogp.cnb.csic.es/tools/venny/) (accessed on 12 December 2022) to obtain the LY- and CUR-BPH target intersection. The relationship between the targets was established using the STRING database (https://string-db.org/) (accessed on 12 December 2022), and the network characteristics of the LY- and CUR-target association network were analyzed using Cytoscape 3.7.2. Finally, the cross-targets were analyzed, and a pathway enrichment diagram was generated using the KEGG database.

### 3.8. Statistical Analysis

Quantitative data were reported as means ± SD and subjected to statistical analysis. Statistical significance was assessed using one-way analysis of variance (ANOVA) followed by Tukey’s comparison test, with GraphPad Software (version 6.02 for windows, San Diego, CA, USA) employed for this purpose. A *p*-value of less than 0.05 was deemed statistically significant. The figures display *p*-values for comparisons between treatment groups and the BPH group, with *, *p* < 0.05; **, *p* < 0.01; ***, *p* < 0.001. *p*-values for comparisons between the BPH group and the CON group are also presented on the figures, with #, *p* < 0.05; ##, *p* < 0.01; ###, *p* < 0.001.

The combination index (CI) was calculated to determine the nature of the LY and CUR combinations based on the methods described with appropriate modifications [32,33]. In brief, the expected value of the combination effect between treatment 1 and treatment 2 was calculated as [(observed treatment 1 value)/(control value)] × [(observed treatment 2 value)]/(control value)] × (control value); and the CI was calculated as the ratio of (observed value of the combination effect)/(expected value of the combination effect). The CI values of <1, >1 and =1 indicate a synergistic, an antagonistic and an additive effect, respectively.

## 4. Discussion

In this work, we demonstrated (1) that the combination effectively attenuated BPH development in rats through the quantitative analysis of histopathology and the prostate index, (2) that the combination treatment decreased the production of both dihydrotestosterone (DHT) and 5α-reductase, (3) that inflammatory responses can be greatly reduced via combination therapy in the BPH model of rats and (4) that AKT1, TNF, EGFR, STAT3 and PTGS2 are main targets which are associated with the anti-BPH activity of lycopene and curcumin.

Although BPH is prevalent among elderly men worldwide, its pathogenesis remains unclear, impeding the advancement of efficacious and secure treatments. The potential mechanisms underlying pathogenesis include systemic/local hormonal and vascular changes that are age-related, as well as the imbalance of cell apoptosis and proliferation and persistent inflammation [34]. Increasing evidence suggests that inflammation may have a key role in BPH development and progression. Inflammation is frequently accompanied with the infiltration of inflammatory cells in prostates, and the infiltrated cells produce cytokines, which may stimulate local growth factor production and angiogenesis [35].

Indeed, four weeks of the oral combination of LY and CUR administration showed promising effects in inhibiting BPH in rats. In particular, combined treatment nearly normalized the prostatic weight and index compared with the BPH group with few side effects. In addition, the inhibition in the progression of BPH in the COM group was more effective than in the LY and CUR group alone. Histologically, the combination treatment was able to significantly improve the prostatic structural organization and reduce fibrotic tissue formation.

**Figure 10:**
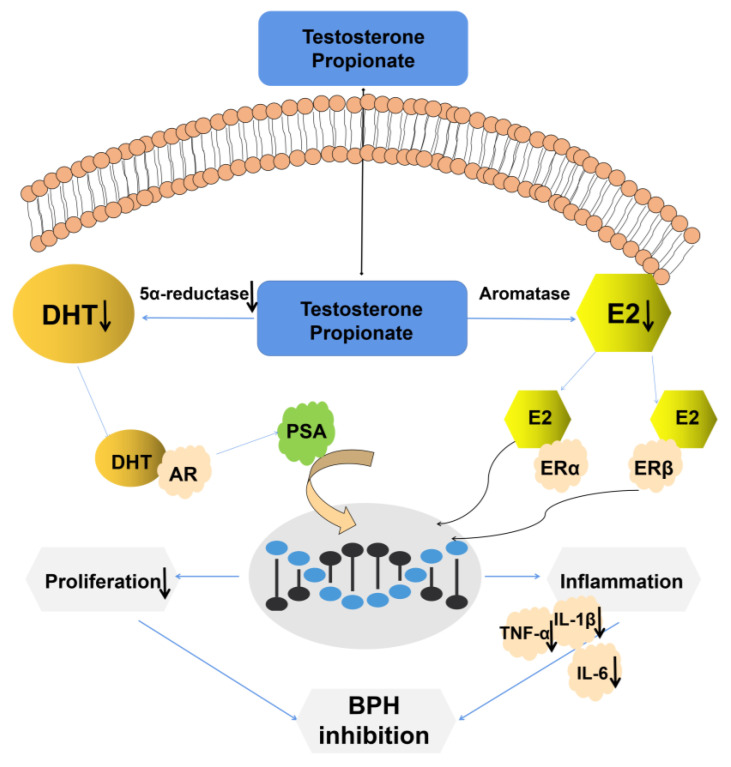
Molecular mechanism diagram.

In this research, we found that the combination of LY and CUR can effectively reduce the production of DHT, 5α-reductase, E2 and PSA (Figure 10). Disordered hormone production is a widely recognized risk factor for BPH due to the crucial role played by hormones such as estrogen, testosterone and DHT in regulating cellular proliferation and apoptosis in the prostate gland. DHT exhibits a 3-fold higher affinity for androgen receptors (ARs) than testosterone, which is converted to DHT via 5α-reductase activity. The serum concentration of DHT has been found to be positively correlated with the incidence and progression of BPH [36,37]. E2, a metabolite of T, is synthesized by the enzyme aromatase and is expressed in the urogenital tract along with fat [38]. Studies have demonstrated a positive correlation between elevated serum estrogen levels or an increased estrogen/androgen ratio and the development of BPH [39].

BPH is significantly associated with chronic inflammation during the aging process. A study demonstrated that the histopathological analysis of 3942 BPH cases revealed a prevalence of 43.1% of inflammatory features, primarily characterized by chronic and mild inflammation [40]. The presence of T lymphocytes and macrophages within the prostate gland leads to the upregulation of cell growth factors and inflammatory cytokines in cases of BPH [41]. Inflammatory factors like interleukin (IL)-6, IL-1α and IL-1*β* may be secreted by the infiltrated lymphocytes, which can stimulate both epithelial and stromal cell proliferation [42,43]. In this research, the inflammatory factors including TNF-α, IL-6 and IL-1β were both significantly reduced by the combined treatment.

The network pharmacology analysis identified 52 matched targets among the predicted targets of LY, CUR and the osteoporosis-associated targets, 12 targets for LY and 40 targets for CUR, which proves that LY and CUR inhibit the progression of BPH via different targets, and their combined treatment covers more comprehensive targets, and thus can improve the therapeutic efficiency. In the PPI network, AKT1 and TNF are the top-ranked genes. AKT1 plays a crucial role in cellular proliferation and viability, exhibiting expression in a diverse range of tissues [44]. TNF primarily facilitates the adhesion and migration of inflammatory cells through upregulating the expression of cell adhesion factors, thereby exerting a crucial influence on the onset and progression of prostatic inflammation. According to Mostafa et al., the passive expansion of peripheral blood vessels facilitates the acquisition of adequate oxygen and nutrients by tissues, thereby promoting prostate cell proliferation and ultimately leading to the development of BPH [45]. Further pathway analysis suggested that the LY and CUR combination’s inhibition of BPH may be in part through regulating pathways in cancer.

In conclusion, we have demonstrated that combined (LY and CUR) treatment can synergistically inhibit the progression of BPH by reducing the expression of inflammatory factors and cell proliferation marker Ki-67 and regulating the hormone level. Additionally, the network pharmacology analysis suggests that the combination of LY and CUR can cover more comprehensive targets and thus improve the therapeutic efficiency. Moreover, it is also important to improve the targeting and bioavailability of drugs in the future due to the poor water solubility and bioavailability of curcumin. Nanotechnology like lipid-based nanoparticles, exosomes and carrier-free nanodrugs would be the focus of our future research [46,47,48,49]. Additionally, translational trials are further required to determine if the combination of LY and CUR is indeed beneficial and more effective for BPH patients.

## Figures and Tables

**Table 1:** Common targets of BPH-LY and CUR interconnection.

No.	Target	Common Name	Uniprot ID
1	Androgen receptor	AR	P10275
2	Estrogen receptor alpha	ESR1	P03372
3	Cytochrome P450 19A1	CYP19A1	P11511
4	Estrogen receptor beta	ESR2	Q92731
5	Vitamin D receptor	VDR	P11473
6	Retinoic acid receptor beta	RARB	P10826
7	MAP kinase ERK2	MAPK1	P28482
8	MAP kinase p38 alpha	MAPK14	Q16539
9	Histone deacetylase 1	HDAC1	Q13547
10	Dual-specificity phosphatase Cdc25A	CDC25A	P30304
11	Peroxisome proliferator-activated receptor alpha	PPARA	Q07869
12	Retinoic acid receptor alpha	RARA	P10276
13	Epidermal growth factor receptor erbB1	EGFR	P00533
14	Serine/threonine-protein kinase AKT	AKT1	P31749
15	Serine/threonine-protein kinase B-raf	BRAF	P15056
16	Apoptosis regulator Bcl-2	BCL2	P10415
17	Signal transducer and activator of transcription 3	STAT3	P40763
18	Cyclooxygenase-2	PTGS2	P35354
19	TNF-alpha	TNF	P01375
20	Tyrosine-protein kinase JAK2	JAK2	O60674
21	Matrix metalloproteinase 9	MMP9	P14780
22	Histone acetyltransferase p300	EP300	Q09472
23	Serine/threonine-protein kinase RAF	RAF1	P04049
24	Matrix metalloproteinase 14	MMP14	P50281
25	Estradiol 17-beta-dehydrogenase 3	HSD17B3	P37058
26	DNA topoisomerase II alpha	TOP2A	P11388
27	Matrix metalloproteinase 7	MMP7	P09237
28	Alkaline phosphatase, tissue-nonspecific isozyme	ALPL	P05186
29	Serine/threonine-protein kinase Aurora-A	AURKA	O14965
30	Glycogen synthase kinase-3 beta	GSK3B	P49841
31	Ribosomal protein S6 kinase 1	RPS6KB1	P23443
32	Nuclear factor erythroid 2-related factor 2	NFE2L2	Q16236
33	Matrix metalloproteinase 13	MMP13	P45452
34	Tyrosine-protein kinase JAK1	JAK1	P23458
35	Arachidonate 5-lipoxygenase	ALOX5	P09917
36	Carbonic anhydrase IX	CA9	Q16790
37	Cyclooxygenase-1	PTGS1	P23219
38	Toll-like receptor (TLR7/TLR9)	TLR9	Q9NR96
39	Plasminogen activator inhibitor-1	SERPINE1	P05121
40	Interleukin-8 receptor B	CXCR2	P25025
41	Macrophage colony stimulating factor receptor	CSF1R	P07333
42	Sphingosine kinase 1	SPHK1	Q9NYA1
43	Type-1 angiotensin II receptor (by homology)	AGTR1	P30556
44	HERG	KCNH2	Q12809
45	ADAM17	ADAM17	P78536
46	Matrix metalloproteinase 3	MMP3	P08254
47	Dipeptidyl peptidase IV	DPP4	P27487
48	Tyrosinase	TYR	P14679
49	Aminopeptidase N	ANPEP	P15144
50	11-beta-hydroxysteroid dehydrogenase 1	HSD11B1	P28845
51	Carbonic anhydrase II	CA2	P00918
52	15-hydroxyprostaglandin dehydrogenase [NAD+]	HPGD	P15428

**Table 2:** Experimental groups.

Group	LY mg/(kg.d) BW	CUR mg/(kg.d) BW	FN mg/(kg.d) BW	Oilve Oil (mL)
(i) CON	0	0	0	1
(ii) BPH	0	0	0	1
(iii) FN	0	0	5	1
(iv) LY	12.5	0	0	1
(v) CUR	0	2.4	0	1
(vi) COM	12.5	2.4	0	1

## Data Availability

The data used to support the findings of this study are available from the corresponding author upon request.

## Abbreviations

BPH	benign prostatic hyperplasia
TP	testosterone propionate
LY	lycopene
CUR	curcumin
FN	finasteride
COM	combination
CON	control
H&E	hematoxylin and eosin
DHT	dihydrotestosterone
IL	interleukin
TNF-α	tumor necrosis factor-alpha
IHC	immunohistochemistry
ELISA	Enzyme-linked Immunosorbent Assay
E2	estradiol
PSA	prostate-specific antigen
CCK-8	Cell Counting Kit 8
T	testosterone
